# Relationship Between Atypical Sexual Fantasies, Behavior, and Pornography Consumption

**DOI:** 10.1177/0306624X221086569

**Published:** 2022-04-12

**Authors:** Ewa B. Stefanska, Nicholas Longpré, Hayley Rogerson

**Affiliations:** 1 University of Greenwich, London, UK; 2 Edge Hill University, Ormskirk, UK; 3 University of Roehampton, London, UK

**Keywords:** atypical sexual fantasies, pornography, sexual behaviors, paraphilia

## Abstract

Paraphilia is a condition in which the sexual excitement relies on fantasizing and/or participating in unusual sexual behaviors although the line between “normal” and “abnormal” has been disputed. The project aimed to explore which sexual fantasies and behaviors are common and uncommon in the general population. Furthermore, the relationship between sexual fantasies, sexual behaviors, and problematic pornography consumption was examined. Finally, the impact of gender was assessed. Analyses were conducted on a sample of 139 participants. Correlations were found between fantasies, behaviors, and problematic pornography consumption. Furthermore, gender differences were found for both sexual fantasies and problematic pornography consumption. Finally, multiple regression revealed that age, gender [Men], fantasies, and behaviors were significant predictor of problematic pornography consumption. Those findings are in-line with previous studies which highlighted that the threshold to consider a sexual practice as being abnormal needs to be reconsidered on the basis of self-reported fantasies and behaviors in the general population.

Even though sexual revolution started in the 1960s, and that we, as a society, have become more open about sexuality and sexual behaviors (Copas et al., 2002; Jones, 2002; Wells & Twenge, 2005), one of the aspects of our sexual lives that continues to be a taboo is sexual fantasies. This might be due to an ambiguous line between which fantasies are considered to be “normal” and which are considered “abnormal.” Certain sexual fantasies, depending on their nature and persons or objects involved, can be classified as atypical and in some cases even recognized as a paraphilia. The Diagnostic and Statistical Manual of Mental Disorders fifth edition (DSM-5) defines paraphilia as “Any intense or persistent sexual interest other than sexual interest in genital stimulation or preparatory fondling with phenotypically normal, physically mature, consenting human partners” (American Psychiatric Association, 2013, p. 685).

The DSM-5 distinguishes between paraphilia and paraphilic disorders, where the latter can be seen as a more severe form of paraphilia, that is, “currently causing distress, impairment to the individual or a paraphilia whose satisfaction has entailed personal harm, or risk of harm, to others” (APA, 2013, p. 685). Paraphilic disorder was introduced in the last iteration of the DSM to distinguish between those who are having atypical sexual practices without having a pathology (Blanchard, 2013) to those who are suffering from it (i.e., clinical distress) or that involving nonconsenting practices. Where treatment is concerned, a paraphilia alone does not necessarily call for clinical intervention, whereas a paraphilic disorder does. Paraphilic disorders include: Voyeurism, Exhibitionism, Frotteurism, Masochism, Sadism, Pedophilia, Fetishism, and Transvestism.

## Are Atypical Sexual Fantasies and Paraphilia More Common Than We Think?

The Kinsey et al. (1948, 1953) reports are seen as pioneers in studying sexuality from a non-medical approach. Gathering interviews from the general population, the findings of the reports highlighted a much higher rate of homosexuality and homosexual experiences than had previously been thought, with 37% of males and 13% of females having experienced at least one orgasm from a homosexual experience. Findings also showed that 12% of females and 22% of males reported an erotic response to a sadomasochistic story, and that 26% of females and 26% of males had frequent erotic responses to biting.

Despite receiving criticism for the sample and analyses used, the Kinsey reports also brought to light the idea that sexual activities that seem to be abnormal may in fact be more common. Moreover, it opened up the conversation on sexuality, leading the way for a plethora of new research on sexuality in the general population (Chiang, 2008). Kinsey et al.’s (1948, 1953) results continue to find support in more recent literature as paraphilia and sexual fantasies are consistently found to be more common than expected (Ogas & Gaddam, 2011). For example, Joyal et al. (2015) investigated sexual fantasies within the general population to understand which sexual fantasies can be noted as rare, unusual, common, or typical. Interestingly, only two sexual fantasies (having sex with a child under 12 years of age and having sex with an animal) were classified as rare. Contrastingly, 30 sexual fantasies were found to be common and 5 were classified as typical, raising a possibility that caution must be taken when labeling a sexual fantasy as abnormal or unusual. Joyal et al. (2015) further argued that because many of sexual fantasies are common, rather than focusing on the prevalence and their content, more research should be conducted investigating the effects of sexual fantasies. This line of inquiry would allow to identify whether some sexual fantasies could be considered potential risk factors for the development of paraphilia, paraphilic disorders, atypical behavior, or potentially harmful sexual behavior.

Abel et al. (1988) highlighted another substantial finding regarding paraphilic behavior by noting that individuals who engage in one type of paraphilia are likely to also engage in other types. In fact, Abel et al.’s (1988) results highlighted that it is rare to find individuals with only 1 paraphilia while most “paraphiliacs” in their sample experienced up to 10 different types of paraphilia. These findings were also supported by Dawson et al. (2016) whose research detected comorbidity among paraphilia’s. Specifically, the authors noted high correlations between many paraphilia sub-scales, suggesting that if an individual has one paraphilia it is likely that they also have symptoms of another paraphilia. It is therefore possible that if an individual is having a number of different paraphilia’s or atypical sexual fantasies, it is also possible that he is engaging in a variety of atypical sexual behaviors, which could in turn increase the likelihood of developing a paraphilic disorder.

## What Gender Differences Can Be Observed in Sexual Fantasies and Paraphilia?

One of the largest-scale studies investigating gender differences in relation to sexual behaviors and fantasies in the general population was “A Billion Wicked Thoughts” by Ogas and Gaddam (2011). The authors looked at online preferences between genders and revealed that whilst men are aroused visually and mostly from single cues, women need stimulation of the mind and numerous cues to feel aroused. This arousal takes place instantly for men after viewing something they find sexually arousing, whilst women take longer to become aroused, favoring a sexually exciting story than any visual triggers. One of the possible hypotheses could be the fact that the physical and psychological arousal for men are integrated, whereas for women physical and psychological arousal are separate, meaning that for women it takes more stimulation simultaneously from different cues to become aroused. Ogas and Gaddam (2011) also indicates that men are more likely to desire the sexual act whereas women are more likely to seek out being desired. As such, women require more emotional, psychological, and intellectual stimulation in order to become aroused, in comparison to men, highlighting the fundamental differences between men and women when it comes to sex, desire, and sexually stimulating materials (Ogas & Gaddam, 2011). It would be therefore possible to hypothesize that there would also be significant gender differences within sexual fantasies, paraphilia, and sexual behavior.

Similarly, Chan (2021a) examined gender differences in paraphilia in a non-clinical sample also noted that males reported significantly more general paraphilic interests, whereas females reported noted significantly more transvestic fetishism. Further, Dawson et al. (2016) found that males showed significantly less repulsion to the majority of paraphilic interests in comparison to women, and that more men reported arousal to most of the paraphilic interests than women did, raising a possibility that males are more likely than females to have a paraphilia. In line with Ogas and Gaddam (2011), Dawson et al. (2016) argue that these gender differences can only be explained by differences in sex drive. This is due to the fact that those who have a low sex drive, namely females, will only use their sexual energy on their preferred sexual targets and activities as it is limited, whereas those with a high sex drive, males, will focus their energy on atypical targets and activities as well as their preferred sexual targets, because they have more sexual energy to release. However, this explanation is controversial, as self-reported sex drive is influenced by social and cultural factors, which in turn influences what is reported versus what people really do behind the closed doors (Baumeister et al., 2001).

Due to these potential differences across gender, it is therefore possible that we might expect differences in paraphilic interests between males and females, with males more likely to engage in a paraphilia as a result of their higher sex drive. However, as such line of thought has not been directly examined, it may also be possible that observed differences between male and female can be explained by bio-psycho-social factors, including a possible difference in sex drive, rather than one lone factor. This nevertheless highlights the need to explore the topic in more depth, further examining how and why atypical sexual fantasies and paraphilia develop in males and females.

## Is There a Link Between Pornography and Paraphilia?

In contrast to paraphilia, pornography is a topic that has been investigated in abundance. The term “pornography” refers to media that are designed to cause sexual excitement by showing or describing sexual acts. Pornography is a part of everyday life for many individuals and the consumption of pornography is large-scale, with PornHub releasing statistics that in 2017 the site received 81 million visits per day, estimating that 75% of viewers are men (Silver, 2018). Although pornography can have some positive effects, it also has negative effects on an individual and on society. Existing research shows links between pornography and aggression, impulse gratification, difficulties in intimate relationships, and many more factors (e.g., Duffy et al., 2016; Love et al., 2015; Poulsen et al., 2013).

A majority of men are more excited by images of submissive and distressed women than by images of smiling and cooperative women (Heilbrun & Leif, 1988). This could explain why an important proportion of pornographic material focuses on contention and domination (Dietz & Evan, 1982). A content analysis of popular pornographic videos carried out by Bridges et al. (2010) noted that 88.2% of the videos contained physical aggression. Common themes included gagging and slapping with mostly male aggressors and female targets. Further it was found that the targets of these acts largely responded with pleasure. These themes are coherent with elements of masochism and sadism and indicate a preference in the general population for pornography with potentially paraphilic elements. Findings from the Bridges et al. (2010) study also showed increasing numbers of popular pornography containing unusual sexual practices, as well as potentially harmful acts. These research findings suggest several conclusions, firstly that pornography involving aggressive behavior is popular and frequent. Secondly, that pornography containing acts that could be considered to be paraphilic is also popular. The success of *Fifty shades of Grey* has highlighted how paraphilic practices can become mainstream and has influenced the onset of these behaviors in the general population (Bonomi et al., 2014).

Few studies have investigated the relationship between pornography and paraphilia. Fisher et al. (2013) have tried to clarify the link between pornography, sexual violence, and paraphilia, specifically pedophilia. Findings from their meta-analysis suggested that despite increasing access to all types of sexually explicit materials, there has been no increase in rates of sexual crimes. Findings from the study also suggested that the influence of pornography on sexual aggression and paraphilia may be regulated by certain predispositions such as history of abuse, promiscuous approach to sexual activity and sexual gratification from control or domination. Therefore, it appears that a mixture of predisposing factors as well as consumption of pornography might correlate with sexually aggressive behavior. These results are in-line with other research on hypersexuality and sexual violence (e.g., Kingston et al., 2017; Knight & Sims-Knight, 2006; Långström & Hanson, 2006). Further, although Fisher et al. (2013) found a link between consumption of child pornography and sexual abusive behavior toward a child, the relationship between pornography consumption and pedophilic paraphilia was unclear. It is therefore difficult to determine a more in depth understanding of the relationship between pornography and paraphilia.

## The Present Study

The aim of the present project is to study the relationship between sexual fantasies, sexual behaviors, and pornography consumption. More specifically, this project aims to (1) explore what sexual fantasies and behaviors are common and uncommon in the general population; (2) how pornography consumption is linked to atypical sexual fantasies and sexual behaviors; and (3) how gender influences sexual fantasies and behaviors, and their relationship to pornography consumption. As such, understanding of the extent of atypical sexual fantasies and behaviors prevalence in the general population (and its potential relationship with problematic pornography consumption) will be considered. The current study will build upon existing research in the area and is proposing to fill some of the gaps in our understanding of paraphilias, providing the field with a necessary update on the topic. It is hypothesized that: (1) Men will report more sexual fantasies, sexual behaviors, and will have more problems with pornography consumption than women; (2) While the prevalence of sexual fantasies and behaviors that seem to be abnormal will be more common, items concerning illegal sexual behavior will not be frequently endorsed by both genders; (3) A significant relationship between pornography consumption, sexual fantasies, and sexual behaviors will be found. It is important to have an awareness of normophilic and paraphilic sexual interests. This is because although having a paraphilia does not appear to increase the risk of sexual reoffending (Brouillette-Alarie et al., 2017; Eher et al., 2016), deviant sexual fantasy is a risk factor (Brankley et al., 2021; Hanson et al., 2007).

## Methods

### Participants

The original sample was composed of *N* = 179 were recruited in the general population through social media and Amazon Mechanical Turk (MTurk). All participants were located in the United Kingdom. Participants were solely sourced from the United Kingdom due to differences in legislation and definitions within the study across countries. Of the original sample, 40 participants were withdrawn because their answers contained too many missing answers. Analyses were conducted on the final sample of *n* = 139. The mean age of participants was 28.41 years old (*SD* = 8.89; range 18–56 years old). The majority of participants were female (*n* = 83; 59.9%), Caucasian (*n* = 115; 82.7%), and in a relationship (*n* = 67; 48.2%). For more details, see Table 1.

**Table 1.** Demographic Characteristics by Frequency and Percentage for the Study Sample.

	Mean	*SD*
Age in years	28.41	8.89
	*N*	%
Gender		
Male	56	40.3
Female	83	59.7
Ethnicity		
Caucasian	115	82.7
Black	2	1.4
Asian	13	9.4
Prefer not to say	3	2.2
Other	6	4.3
Marital status		
Single	67	48.2
Married/living with a partner	67	48.2
Divorced	5	3.6
Religion		
Christian	42	30.2
Buddhist	1	0.7
Muslim	4	2.9
Hindu	4	2.9
Jewish	1	0.7
No religion	76	54.7
Other	11	7.9
Level of education		
GCSE	15	10.8
A-level/Btec	40	28.8
Undergraduate degree	53	38.1
Postgraduate degree	31	22.3

### Procedures

The project has received ethical approval by a university in England and Wales. The survey was created on and administered using Qualtrics (Qualtrics^®^, Provo, UT). Qualtrics is an online survey software tool used to create and distribute surveys and analyze responses. It also allowed for anonymity as respondents and researchers are never in contact.

Participants were sourced through the use of social media (Facebook, Instagram, and Snapchat) and MTurk. A pilot was first conducted on social media (*n* = 25) to ensure instructions were clear, to identify potential problems in the survey and to examine participants’ willingness to take part. The survey was then distributed via MTurk, an online crowdsourcing space that allows both individuals and businesses to source candidates that are willing to respond to surveys and tasks in exchange for payment. MTurk allows individuals to source a workforce remotely, and if necessary, from specific locations anywhere in the world. All participants were required to consent in order to participate in the research study, and if consent was not given the surveys were skipped and the participant was immediately presented with the concluding screen thanking them for their time. The duration of the study lasted between 25 and 45 minutes.

### Materials

For the purpose of this study, three scales were used:

#### The Amended Wilson Sex Fantasy Questionnaire (SFQ)

The amended version of the SFQ, developed by Joyal et al. (2015), was used in order to determine both the nature and intensity of participants’ sexual fantasies (Wilson & Lang, 1981). The SFQ is composed of 55 statements that participants rated using a 5-point Likert-style scale (ranging from 0—“Never” to 4—“Always.” An example of an item is “I have fantasized about tying someone up in order to obtain sexual pleasure.” Participants were asked to indicate how often each of the statements applied to them. Concluding the questionnaire was one open question; “If your favorite sexual fantasy was not included in the questionnaire, please write it down here”: this was in order to see the nature of each participant’s preferred sexual fantasy. In the present study, the Cronbach’s alpha was .96.

#### The Sexual Behavior Questionnaire (SBQ)

The amended version of the SFQ (Joyal et al., 2015) was used to create the SBQ. In the current study, every statement was changed from fantasy to behavior. For example, “Atmosphere and location are important in my sexual fantasies” was changed to “Atmosphere and location are important aspects of my sexual relationships.” The SBQ is composed of 55 statements that participants rated using a 5-point Likert-style scale (ranging from 0—“Never” to 4—“Always.” An example of an item is “I have willingly tied up a sexual partner and gained sexual pleasure from this.” Participants were specifically asked to think about both past and present in order to obtain a comprehensive view of their sexual behaviors. In the present study, the Cronbach’s alpha was .95.

#### The Problematic Pornography Consumption Scale (PPCS-18)

The PPCS is composed of 18 statements used to measure pornography consumption through six factors: (1) salience, (2) mood modification, (3) conflict, (4) tolerance, (5) relapse, and (6) withdrawal (Bőthe et al., 2018). This scale was used in order to distinguish between individuals who have non-problematic pornography use and individuals who have problematic pornography use. The scale is rated on a 7-point Likert-style scale (ranging from 1—“Never” to 7—“All the time”) based on how often or to what extent each statement applied to them. An example of an item is “I became stressed when something prevented me from watching porn.” Studies have shown good reliability and validity across samples, countries, and gender (Bőthe et al., 2018; Burdis et al., 2022; Chen et al., 2021). In the present study, the Cronbach’s alpha was .96.

### Analyses

First, power analyses were conducted with G*Power software to determine if the final sample size was sufficient to detect the effect of each test at the desired level of significance. Power analyses revealed that a sample between *n* = 40 and 132 was sufficient to uncover large to medium effect size across analyses. Furthermore, distribution of the SFQ, SBQ, and PPCS were tested prior to the analyses. Skewness was ranging from 1.08 to 1.91 and kurtosis was ranging from 1.32 to 1.92. Values between −2 and +2 are considered acceptable and support the normal distribution assumption (George & Mallery, 2010). Thirdly, frequency analyses were conducted to assess the prevalence of fantasies and behaviors. Analyses were conducted on the men and the women sub-sample. To reduce the problem of low probability response choices, the five-point scales were collapsed into three-point scales; the amended version of the SFQ and the SBQ were recoded from 1–5 to 0–3 (1 became 0 [Absent], 2 and 3 became 1 [Mild], 4 became 2 [Moderate], and 5 became 3 [Strong]). This procedure was used in previous studies on paraphilias (e.g., Burdis et al., 2022; Joyal et al., 2015; Kingston et al., 2017; Longpré et al., 2020) and has produced stable and consistent results. Secondly, *t*-tests were conducted to assess gender differences on the three scales. Then, Pearson’s Correlation Coefficient was conducted to investigate the relationships between scales. Finally, multiple linear regressions were performed to predict the level of problematic pornography consumption. All statistical analyses were carried out using the Statistical Package for Social Sciences (SPSS) Version 26 (IBM, New York, USA).

## Results

First, frequency analyses were conducted to assess the prevalence of fantasies and behaviors among men and women. Results are presented in Table 2; fantasies and behaviors that are associated to a category of paraphilias are highlighted in bold. Women are reporting more fantasies and behaviors related to being dominated (Women [*F* = 48.7%; *B* = 34.9%]; Men [*F* = 19.2%; *B* = 12.5%]), being restrained (Women [*F* = 38%; *B* = 23.2%]; Men [*F* = 15.1%; *B* = 16.1%]) or being spanked (Women [*F* = 20.4%; *B* = 30.1%]; Men [*F* = 9.4%; *B* = 14.5%]). Men are reporting more fantasies and behaviors related dominating someone (Women [*F* = 20.3%; *B* = 20.5%]; Men [*F* = 34%; *B* = 26.8%]) or spanking someone (Women [*F* = 11.4%; *B* = 13.3%]; Men [*F* = 15.1%; *B* = 16.5%]). Fantasies and behaviors related to voyeurism, fetichism, exhibitionism, transvestism, sadism, and urophilia were similar across gender. A small number of participants reported pedophilic (Women [*F* = 3.8%; *B* = 4.8%]; Men [*F* = 1.9%; *B* = 1.8%]) or zoophilic (Women [*F* = 5.1%; *B* = 2.4%]; Men [*F* = 1.9%; *B* = 1.8%]) fantasies and behaviors.

**Table 2.** Fantasies and Behaviors Frequency for the Study Sample.

	Fantasy (moderate/strong)		Behavior (moderate/strong)	
	Men (%)	Women (%)	Men (%)	Women (%)
I have [..] about romantic emotions during a sexual relationship.	77.40	75.90	71.40	75.90
I have [..] about taking part in fellatio/cunnilingus.	43.40	39.70	58.10	59.80
I have [..] about having sex in an unusual place (e.g., in the office; public toilets).	22.70	25.30	10.70	14.60
I have [..] about atmosphere and location	20.20	46.20	41.80	56.10
I have [..] about having sexual in a romantic location (e.g., on a deserted beach).	37.70	33.30	14.30	16.90
I have [..] about having sex with someone that I know who is not my spouse	39.60	21.50	21.40	13.30
I have [..] about masturbating my partner.	43.40	31.60	66.10	57.80
I have [..] about being masturbated by my partner.	53.80	38	62.50	56.60
I have [..] about having sex with two women.	39.60	8.90	8.90	2.40
I have [..] about watching two women make love.	29.30	6.30	10.70	9.60
I have [..] about having sex with an unknown person.	34	13.90	12.50	7.30
I have [..] about making love openly in a public place.	15.10	15.20	7.10	6
**I have [..] about being dominated sexually.**	19.20	48.70	12.50	34.90
I have [..] about giving cunnilingus.	28.30	16.50	42.90	21.70
I have [..] about having sex with a star or a well-known person.	25.80	25.30	25	24.10
I have [..] about giving fellatio.	20.80	31.60	23.20	50.60
**I have [..] about dominating someone sexually.**	34	20.30	26.80	20.50
I have [..] about being masturbated by an acquaintance.	26.40	13.90	10.70	7.20
**I have [..] about being tied up by someone in order to obtain sexual pleasure.**	15.10	38	16.10	23.20
I have [..] about masturbating an acquaintance.	24.50	13.90	14.30	4.80
I have [..] about being masturbated by an unknown person.	17	20.10	7.10	7.20
I have [..] about having anal sex.	30.20	8.90	14.30	15.90
I have [..] about having sex with more than three people, all women.	28.30	6.30	3.40	4.80
I have [..] about masturbating an unknown person.	23.60	6.30	5.50	3.70
**I have [..] about tying someone up in order to obtain sexual pleasure.**	11.30	21.50	7.30	15.70
**I have [..] about watching someone undress without him or her knowing.**	18.90	10.10	3.60	3.60
I have [..] about having interracial sex.	22.60	22.70	14.30	14.60
I have [..] about having sex with a woman with very large breasts.	22.60	8.90	33.90	4.80
I have [..] about ejaculating on my sexual partner. (For men only)	41.50	NA	49.10	N/A
I have [..] about having sex with someone much older than me.	24.50	19	14.50	12
I have [..] about having sex with more than three people, both men and women.	13.20	10.10	3.60	2.40
I have [..] about having sex with two men.	5.70	15.20	3.60	4.80
I have [..] about being photographed or filmed during a sexual relationship.	9.40	6.30	8.90	4.80
I have [..] that my partner ejaculates on me.	9.40	19.00	12.50	36.10
I have [..] about having sex with someone much younger (legally) than me	26.40	6.30	8.90	6.10
I have [..] about petting with a total stranger in a public place (e.g., metro).	9.40	2.50	7.10	3.60
I have [..] about indulging in sexual swinging with a couple that I do not know.	11.50	6.30	1.80	1.20
**I have [..] about spanking or whipping someone to obtain sexual pleasure.**	15.10	11.40	16.50	13.30
**I have [..] about being spanked or whipped to obtain sexual pleasure.**	9.40	20.40	14.50	30.10
I have [..] about having homosexual (or gay) sex.	11.80	21.50	8.90	13.30
I have [..] about having a sexual relationship with a woman with very small breasts.	24.50	3.80	23.20	6.10
I have [..] about indulging in sexual swinging with a couple that I know.	11.30	3.80	3.60	4.80
I have [..] about being forced to have sex.	9.40	16.50	3.60	7.20
**I have [..] about having sex with a fetish or non-sexual object.**	7.50	8	3.60	2.40
I have [..] about having sex with a prostitute or a stripper.	11.30	5.10	5.40	3.60
I have [..] about having sex with more than three people, all men.	3.80	7.60	3.60	3.60
**I have [..] about showing myself naked or partially naked in a public place.**	5.70	6.40	3.60	3.70
**I have [..] about watching two men make love.**	3.80	11.40	3.60	3.60
**I have [..] about sexually abusing a person who is drunk, asleep, or unconscious.**	5.70	3.80	7.10	3.60
**I have [..] about forcing someone to have sex.**	3.80	4.10	5.40	4.80
**I have [..] about wearing clothing associated with the opposite sex.**	1.90	4.10	3.60	8.40
**I have [..] about my sexual partner urinating on me.**	7.50	2.50	1.80	1.20
**I have [..] about urinating on my sexual partner.**	1.90	3.80	1.80	4.80
**I have [..] about having sex with an animal.**	1.90	5.10	1.80	2.40
**I have [..] about having sex with a child under the age of 12 years.**	1.90	3.80	1.80	4.80

Second, Pearson’s moment correlations were conducted. Results are presented in Table 3. Fantasies was correlated with behaviors (*r* = .83, *p* < .001) and problematic pornography consumption (*r* = .67, *p* < .001). Behaviors was also correlated with problematic pornography consumption (*r* = .65, *p* < .001)

**Table 3.** Sub-Scales Correlations.

	SFQ	SQB	PPCS
SFQ	1	.834***	.696***
SQB		1	.654***
PPCS			1

*** *p* < .001.

Third, Student’s *t*-tests were conducted to assess the impact of gender (men/women) on the score of each scale. Results are presented in Table 4. Men reported more fantasies (*t*(137) = 2.55, *p* = .012) and problematic pornography consumption (*t*(137) = 5.86, *p* < .001) than women. However, no differences were found between men and women for the number of behaviors.

**Table 4.** Gender Differences for the Scales Used in the Study.

	Men (*n* = 56)	Women (*n* = 83)	*t*
SFQ	44.75 (24.77)	33.81 (23.83)	2.55**
SQB	34.89 (21.33)	29.39 (20.40)	1.53
PPCS	46.68 (22.75)	27.48 (15.89)	5.86***

** *p* < .01. ***p < .001.

Finally, linear regressions were conducted to assess which variables predict problematic pornography consumption. Results are presented in Table 5. Results indicated that age (*b* = 0.12, *t*(125) = 2.07, *p* = .040), gender (*b* = .31, *t*(125) = 5.22, *p* < .001), fantasies (*b* = 0.41, *t*(125) = 3.85, *p* < .001), and behaviors (*b* = 0.28, *t*(125) = 2.66, *p* = .009) significantly predict problematic pornography consumption. These variables explained a significant proportion of variance, *R*^2^ = .62, *F* (4) = 48.43, *p* < .001.

**Table 5.** Variables Predicting Problematic Pornography Consumption.

	*b*	*t*
Age	0.12	2.07*
Gender (1 = men)	0.31	5.22***
SFQ	0.41	3.85***
SBQ	0.28	2.66**

* *p* < .05. **p < .01. ***p < .001. *R*^2^ *=* .62 (*N* = 139, *p* < .0001).

## Discussion

To date, literature concerning sexual fantasies has focused mostly on the prevalence of fantasies and the extent of gender differences within these fantasies. Most of the research that has been conducted has investigated one or two concepts, for example, sexual fantasies and gender or pornography and sexual fantasies. However, to our knowledge only few studies have explored the relationships between all three concepts: (1) pornography consumption, (2) sexual fantasies, and (3) sexual behaviors. This has left gaps in the research with crucial information lacking about how these atypical sexual fantasies develop and their effects on the psychosocial development. Therefore, the current research aimed to examine the relationships between all of these three factors, alongside gender differences, allowing for a more comprehensive picture related to sexual fantasies.

The results showed that pornography consumption, sexual fantasies, and sexual behaviors were positively correlated at a statistically significant level. The strongest correlation was found between sexual fantasies and sexual behaviors, indicating that those who have more sexual fantasies engage in more sexual behaviors. This finding is not surprising as we would expect that individuals who have more sexual fantasies would potentially be more willing to participate in more sexual behaviors due to the natural progression from fantasizing to acting out fantasies (Longpré et al., 2019). Chan (2021b) also found that individuals who have more paraphilic interests and more likely to engage in sexual risk-taking behavior. The results also found a moderate and positive correlation between sexual fantasies and pornography consumption, suggesting that those with a more problematic relationship with pornography have more sexual fantasies. It is important to note that for those scoring higher on the Problematic Pornography Consumption Scale, this does not necessarily mean they watch a high volume of pornography, it shows that they have a more problematic relationship with pornography. These results support the findings by Paolucci et al. (1997) who noted that exposure to pornography is related to sexually deviant tendencies and an increased risk of committing sexual offences. The current study further suggests that not only is exposure to pornography linked with sexual fantasies, but the more problematic and potentially harmful the relationship an individual has with pornography, the higher the number of sexual fantasies they seem to present. However, while on the one hand problematic pornography consumption could cause an individual to develop more sexual fantasies, on the other hand, it could be that individuals have already developed these sexual fantasies and then began to develop an unhealthy relationship with pornography. Lastly, a moderate positive correlation was also found for the relationship between sexual behaviors and pornography consumption indicating that individuals with a more problematic relationship with pornography experience more sexual behaviors. However, as highlighted by Fisher et al. (2013), access to sexually explicit materials is not directly linked to an increase in rates of sexual crimes. In a recent study by Burdis et al. (2022), mediation analyses were conducted to assess the relationship between Trauma, Paraphilias (Arousal), Problematic Pornography, and Hypersexuality. Analyses revealed that the path (direct effect) between Pornography and Arousal was positive (*b* = 0.224, *SE* = .028, *p* < .001), indicating that problematic relationship with pornography preclude paraphilic arousal. However, the correlational design used in the current study is not allowing to clarify if paraphilic behaviors preclude problematic relationship with pornography or the other way around. Future research should focus on Structural Equation Modeling to clarify the temporal order between paraphilic fantasies, paraphilic behaviors, and problematic relationship with pornography.

When examining items on the sex fantasy questionnaire separately, the analysis is revealing that the items concerning illegal sexual behavior are the least frequently endorsed. In our sample, the most rarely endorsed items were: “I have willingly had sex with a child under the age of 12” (child molesting); “I have willingly had sex with an animal” (zoophilia); and “I have willingly sexually abused someone who was drunk, asleep, or unconscious” (rape). In contrast, the most frequently endorsed items were: “I have willingly participated in a sexual activity where I dominated a sexual partner”; and “I have willingly masturbated on my partner.” These findings parallel the findings from Joyal et al. (2015) who reported the two items that were endorsed the least were *having sex with an animal* and *having sex with a child under 12*. A recent study by Burdis et al. (2022), conducted on a sample from the general population and Fetlife [a social networking website for individuals interested in BDSM, fetishism, and kink], also reported a similar trend. The low prevalence of zoophilia and sexual attraction toward children is consistent across samples. Furthermore, the items classified as typical in our study are also similar to the findings reported by Joyal et al. (2015). The importance of atmosphere, location, oral sex, and consensual domination were frequently endorsed. These results are consistent across samples.

The results examining gender differences showed significant differences between male and female scores for both the Problematic Pornography Consumption scale and the Sex Fantasy questionnaire, with males scoring higher on both scales. However, no significant gender differences were found for the sexual behavior questionnaire. This suggests that male and female behaviors, when it comes to sex, are quite similar (e.g., Ogas & Gaddam, 2011), with more similarities for behaviors than fantasies and pornography consumption. The gender differences for pornography consumption and sexual fantasies are concordant with previous research (e.g., Dawson et al., 2016; Ogas & Gaddam, 2011; Silver, 2018). It is interesting that no significant gender differences were found for the sexual behavior scores, as this suggests that females are likely to engage in just as many sexual behaviors as males. However, they do not fantasize as much as males, nor do they have as much of a problematic relationship with pornography as males do. However, whether or not females welcomed or simply experienced these behaviors are not known as this was not measured. We also do not know if such behaviors were pleasurable or appealing. Although research is limited, the narratives of consent in rough sex indicate that men are more likely to engage in BDSM practices (bondage/discipline, dominance/submission, and sadism/masochism). Indeed, data suggests that for women who have experienced BDSM behaviors, a significant proportion did not consent (Bows & Herring, 2020). The boundaries of BDSM role paly during sadomasochistic sex are known to be sometimes transgressed and enter the realm of physical and/or psychological abuse (Warren & Hazelwood, 2002).

The results from regression analysis showed that sexual fantasies, sexual behaviors, and gender significantly predicted problematic pornography consumption. When combining these findings with the positive correlation results, we can conclude that higher levels of sexual fantasies and sexual behaviors can predict higher levels of problematic pornography consumption. The possibility of sexual progression to acting out on atypical fantasies (and/or to the development of paraphilia) is complex. Although fantasy is universal to the human condition, the process of introduction of stimulus is not clear. A newly encountered object can be discarded, ignored, or rejected, but it can also be welcomed (incorporated out of curiosity) or desired (appearing specifically for erotic arousal). The decision regarding how to process the stimuli does not always occur through reason and can be subconscious (Mellor, 2017). It appears that the primary motivation for sexual fantasies is to stimulate or enhance sexual arousal, not to compensate for state of depravation (Leitenberg & Henning, 1995). However, because sexual fantasies tend to lose their erotic value, they continuously go through minor adaptations in order to maintain its value. This process can lead to deviant content (Laws & Marshall, 1990). In such instance, pornography can not only allow easy access to such content but provide a sense of validation that other people have similar sexual interests (Innala, 2007). Sometimes, indulgence in deviant fantasies or over the internet is insufficient to produce anticipated sexual euphoria. These individuals begin to seek alternatives (Chan et al., 2011) either consciously (planned offence) or unconsciously (opportunistic offence) and eventually act out.

### Implications

One important implication in the present study is the significant relationship between sexual fantasies and sexual behaviors. Although, it is now recognized that people have more sexual fantasies than initially thought, and also more atypical sexual fantasies than first thought (Joyal et al., 2015; Kinsey et al., 1948, 1953; Ogas & Gaddam, 2011), not much research has been conducted investigating possible effects of these fantasies. The relationship between sexual fantasies and sexual behaviors suggests that one may have an effect on the other and it may be the case that having atypical sexual fantasies is a risk factor for the development of atypical sexual behaviors. However, it is of the utmost importance that we remember that this research is correlational, and more research must be conducted to understand how this relationship is working. Thus, this research provides a foundation to use and to build upon. If it is the case that holding atypical sexual fantasies is a risk factor for the development of atypical sexual behavior, we must then consider the risk of subsequent development of a paraphilia or even a paraphilic disorder. While having a paraphilia is not associated to an increase of risk of reoffending (Brouillette-Alarie et al., 2017; Eher et al., 2016), higher levels of risky sexual behaviors along paraphilic interests might be indicative of sexual offending behavior (Chan, 2020, 2021a). Further, deviant sexual fantasy is recognized as a risk factor of sexual reoffending (i.e., deviant sexual interests [STABLE-2007; Brankley et al., 2021; Hanson et al., 2007], sexual preoccupation [ACUTE-2007; Hanson et al., 2007]). Therefore, it is imperative to determine where the line is between typical and atypical sexual fantasies, on the basis of non-arbitrary threshold, and based on empirical findings, not on the basis of judgmental and outdated beliefs. A threshold should be determined by statistical analysis (Longpré et al., 2018; Ruscio et al., 2006), and several studies have shown that the DSM paraphilic categories are failing to respect this important principle (e.g., Burdis et al., 2022; Joyal et al., 2015; Ogas & Gaddam, 2011). That is because too low threshold leads to type I error, stigmatization, and impacts our ability to develop effective prevention and treatment programs.

The present study supports the notion that, when it comes to pornography consumption and reliance on sexual fantasy life, males and females differ in a number of ways. Males reported more problematic relationship with pornography than females did, and males were more likely to report more sexual fantasies than females. This is a very useful finding that should be taken into consideration from etiological and treatment standpoint. Fantasy can have an addictive quality allowing for fun, relaxation, escape from trauma, or boredom (Mellor, 2017). Internet porn, having similar qualities, is also addictive and there is a strong evidence suggesting that at a neuroscientific level internet pornography use is addictive the same way other internet-related behaviors (e.g., gaming) are (Love et al., 2015). This is not to say that just because participants score higher on these scales that they need treatment, but for individuals that have more severe problems with pornography or for those whom a sexual fantasy has turned into a paraphilia or paraphilic disorder treatment may be an option. The significant gender differences that have been found in this study and existing research suggests that when it comes to treatment, different approaches need to be in place to successfully treat a male in comparison with a female. This is something that must be considered when creating treatment programs and is a fact that treatment facilitators should be made aware of in order to provide successful treatment for all genders.

Although more research is needed to understand the relationship between problematic pornographic consumption and sexual fantasies as well as behaviors, we know that pornography use can have a variety of negative consequences (e.g., Paolucci et al., 1997). These include aggression, impulse gratification, and difficulties in intimate relationships, among many others. Though we cannot say that pornography consumption effects sexual fantasies and sexual behaviors directly, findings have shown that there is a relationship between all three variables and that sexual fantasies, gender, and sexual behaviors can significantly predict levels of problematic pornography consumption. We know that not all sexual fantasies and sexual behaviors are harmful and dangerous, in fact the majority of them are not. However, for individuals that engage in potentially harmful, dangerous, or illegal fantasies or behaviors we must consider that having a problematic relationship with pornography is at least known to have a relationship with these factors, harmful or not.

### Limitations and Further Research

This study has its limitations. First, data were obtained through self-reported fantasies and behaviors, which relies on a level of honesty from participants and can be subject to response bias. It is impossible to know which information was disclosed, hidden, or modified, and to what extent. For example, social desirability bias may be a concern when revealing pedophilic sexual interests. However, the present study was voluntary, and anonymous. Furthermore, outside of the minimal financial compensation for the MTurk subsample, no advantages were offered to participants outside of contributing to increase our knowledge on sexuality. Thus, the impact of social desirability was controlled as much as possible for this kind of project. Furthermore, results obtained were similar to previous research (e.g., Joyal et al., 2015; Kinsey et al., 1948, 1953; Ogas & Gaddam, 2011), indicating stability across samples, which indicates a good reliability in our findings.

A second limitation steamed from the sample under scrutiny. Participants were coming from the general population, with a majority of Caucasian females, which might affect the generalizability of the data. However, our results are similar to previous research on this topic, and no unexpected results were obtained. Future study should also include individuals with disclosed sexual interests in BDSM, fetishism, and kink (i.e., Burdis et al., 2022). In order to determine where to draw the line between typical and atypical sexual fantasies/behaviors, and to avoid judgmental threshold, studies need to be conducted and replicated on heterogenous samples that cover the spectrum of sexual fantasies and behaviors.

## Conclusion

Existing research surrounding sexual fantasies focuses on gender differences and frequency of more atypical fantasies whilst research regarding pornography consists mostly of its relationship with sexual offences. This study has provided an updated support for existing findings for gender differences and has contributed new findings showing the relationship between sexual fantasies and behaviors and also highlighting these variables as predictors of problematic pornography consumption. The findings from this study have strengthened existing research and have provided us with a new foundation upon which future research can be built. Future research should consider the new relationship between sexual fantasies and behaviors, possible risk factors for the development of atypical sexual fantasies, the real nature of the relationship between pornography consumption, and both sexual fantasies and behaviors.

## References

[bibr1-0306624X221086569] Abel G. G. Becker J. V. Cunningham-Rathner J. Mittelman M. Rouleau J. L. (1988). Multiple paraphilic diagnoses among sex offenders. Journal of the American Academy of Psychiatry and the Law Online, 16(2), 153–168. 3395701

[bibr2-0306624X221086569] American Psychiatric Association. (2013). Diagnostic and statistical manual of mental disorders (DSM-5^®^). American Psychiatric Publishing.

[bibr3-0306624X221086569] Baumeister R. F. Catanese K. R. Vohs K. D. (2001). Is there a gender difference in strength of sex drive? Theoretical views, conceptual distinctions, and a review of relevant evidence. Personality and Social Psychology Review, 5(3), 242–273. 10.1207/S15327957PSPR0503_5

[bibr4-0306624X221086569] Blanchard R. (2013). A dissenting opinion on DSM-5 pedophilic disorder. Archives of Sexual Behavior, 42(5), 675–678. 10.1007/s10508-013-0117-x 23677282

[bibr5-0306624X221086569] Bonomi A. E. Nemeth J. M. Altenburger L. E. Anderson M. L. Snyder A. Dotto I. (2014). Fiction or not? Fifty shades is associated with health risks in adolescent and young adult females. Journal of Women’s Health, 23(9), 720–728. 10.1089/jwh.2014.4782 25144515

[bibr6-0306624X221086569] Bőthe B. Tóth-Király I. Zsila Á. Griffiths M. D. Demetrovics Z. Orosz G. (2018). The development of the problematic pornography consumption scale (PPCS). The Journal of Sex Research, 55(3), 395–406. 28276929 10.1080/00224499.2017.1291798

[bibr7-0306624X221086569] Bows H. Herring J. (2020). Getting away with murder? A review of the ‘rough sex defence.’ The Journal of Criminal Law, 84(6), 525–538. 10.1177/0022018320936777

[bibr8-0306624X221086569] Brankley A. E. Babchishin K. Hanson R. K. (2021). STABLE-2007 demonstrates predictive and incremental validity in assessing risk-relevant propensities for sexual offending: A meta-analysis. Sexual Abuse, 33(1), 34–62. 10.1177/1079063219871572 31516097

[bibr9-0306624X221086569] Bridges A. J. Wosnitzer R. Scharrer E. Sun C. Liberman R. (2010). Aggression and sexual behavior in best-selling pornography videos: A content analysis update. Violence Against Women, 16(10), 1065–1085. 20980228 10.1177/1077801210382866

[bibr10-0306624X221086569] Brouillette-Alarie S. Proulx J. Hanson R. K. (2017). Three central dimensions of sexual recidivism risk: Understanding the latent constructs of Static-99R and Static-2002R. Sexual Abuse, 30, 676–704. 28183223 10.1177/1079063217691965

[bibr11-0306624X221086569] Burdis C. Longpré N. Guay J. P. (2022). The impact of childhood trauma on the development of paraphilic interests. [Manuscript submitted for publication].

[bibr12-0306624X221086569] Chan H. C. O. (2020). The victim-offender overlap in sexual offending: Exploring a community-based sample of young adults in Hong Kong. Sexual Abuse, 33(8), 923–949. 10.1177/1079063220981889 33353485

[bibr13-0306624X221086569] Chan H. C. O. (2021a). Paraphilic interests: The role of psychosocial factors in a sample of young adults in Hong Kong. Sexuality Research and Social Policy, 19, 159–178. 10.1007/s13178-020-00532-z

[bibr14-0306624X221086569] Chan H. C. O. (2021b). Risky sexual behavior of young adults in Hong Kong: An exploratory study of psychosocial risk factors. Frontiers in Psychology, 12, 658179. 10.3389/fpsyg.2021.658179 33828516 PMC8019819

[bibr15-0306624X221086569] Chan H. C. O. Heide K. M. Beauregard E. (2011). What propels sexual murderers: A proposed integrated theory of social learning and routine activities theories. International Journal of Offender Therapy and Comparative Criminology, 55(2), 228–250. 10.1177/0306624X10361317 20160008

[bibr16-0306624X221086569] Chen L. Luo X. Bőthe B. Jiang X. Demetrovics Z. Potenza M. N. (2021). Properties of the Problematic Pornography Consumption Scale (PPCS-18) in community and subclinical samples in China and Hungary. Addictive Behaviors, 112, 106591. 10.1016/j.addbeh.2020.106591 32768797

[bibr17-0306624X221086569] Chiang H. H. H. (2008). Effecting science, affecting medicine: Homosexuality, the Kinsey reports, and the contested boundaries of psychopathology in the United States, 1948–1965. Journal of the History of the Behavioral Sciences, 44(4), 300–318. 18831516 10.1002/jhbs.20343

[bibr18-0306624X221086569] Copas A. J. Wellings K. Erens B. Mercer C. H. McManus S. Fenton K. A. Korovessis C. Macdowall W. Nanchahal K. Johnson A. M. (2002). The accuracy of reported sensitive sexual behaviour in Britain: Exploring the extent of change 1990–2000. Sexually Transmitted Infections, 78(1), 26–30. 11872855 10.1136/sti.78.1.26 PMC1763702

[bibr19-0306624X221086569] Dawson S. J. Bannerman B. A. Lalumière M. L. (2016). Paraphilic interests: An examination of sex differences in a nonclinical sample. Sexual Abuse, 28(1), 20–45. 24633420 10.1177/1079063214525645

[bibr20-0306624X221086569] Dietz P. E. Evans B. (1982). Pornographic imagery and prevalence of paraphilia. American Journal of Psychiatry, 139, 1493–1495. 7137403 10.1176/ajp.139.11.1493

[bibr21-0306624X221086569] Duffy A. Dawson D. L. das Nair R. (2016). Pornography addiction in adults: A systematic review of definitions and reported impact. Journal of Sexual Medicine, 13(5), 760–777. 10.1016/j.jsxm.2016.03.002 27114191

[bibr22-0306624X221086569] Eher R. Schilling F. Hansmann B. Pumberger T. Nitschke J. Habermeyer E. Mokros A. (2016). Sadism and violent reoffending in sexual offenders. Sexual Abuse: A Journal of Research and Treatment, 28, 46–72. 10.1177/1079063214566715 25567533

[bibr23-0306624X221086569] Fisher W. A. Kohut T. Di Gioacchino L. A. Fedoroff P. (2013). Pornography, sex crime, and paraphilia. Current Psychiatry Reports, 15(6), 362. 23636986 10.1007/s11920-013-0362-7

[bibr24-0306624X221086569] George D. Mallery M. (2010). SPSS for Windows Step by Step: A Simple Guide and Reference, 17.0 update (10a ed.). Pearson.

[bibr25-0306624X221086569] Hanson R. K. Harris A. J. R. Scott T.-L. Helmus L. (2007). Assessing the risk of sexual offenders on community supervision: The Dynamic Supervision Project (User report no. 2007–05). Public Safety Canada. http://www.publicsafety.gc.ca/cnt/rsrcs/pblctns/ssssng-rsk-sxl-ffndrs/index-eng.aspx

[bibr26-0306624X221086569] Heilbrun A. Leif D. (1988). Autoerotic value of female distress in sexually explicit photographs. Journal of Sex Research, 24, 47–57. 22375634 10.1080/00224498809551397

[bibr27-0306624X221086569] Innala S. (2007). Pornography on the net: Same attraction, but new options, Sexologies, 16(2), 112–120. 10.1016/j.sexol.2006.12.010

[bibr28-0306624X221086569] Jones R. L. (2002). “That’s very rude, I shouldn’t be telling you that”: Older women talking about sex. Narrative Inquiry, 12(1), 121–143.

[bibr29-0306624X221086569] Joyal C. C. Cossette A. Lapierre V. (2015). What exactly is an unusual sexual fantasy? The Journal of Sexual Medicine, 12(2), 328–340. 25359122 10.1111/jsm.12734

[bibr30-0306624X221086569] Kingston D. A. Graham F. J. Knight R. A. (2017). Relations between self-reported adverse events in childhood and hypersexuality in adult male sexual offenders. Archive of Sexual Behaviors, 46(3), 707–720. 10.1007/s10508-016-0873-5 27752854

[bibr31-0306624X221086569] Kinsey A. Pomeroy W. Martin C. (1948). Sexual behavior in the human male. Saunders. 10.2105/ajph.93.6.894 PMC1447861 12773346

[bibr32-0306624X221086569] Kinsey A. Pomeroy W. Martin C. Gebhard P. (1953). Sexual behavior in the human female. Saunders.

[bibr33-0306624X221086569] Knight R. A. Sims-Knight J. (2006). The developmental antecedents of sexual coercion against women: Testing alternative hypotheses with structural equation modeling. Annals of the New York Academy of Sciences, 989(1), 72–85. 10.1111/j.1749-6632.2003.tb07294.x 12839887

[bibr34-0306624X221086569] Långström N. Hanson R. (2006). High rates of sexual behaviour in the general population: Correlates and predictors. Archives of Sexual Behaviour, 35(1), 37–52. 10.1007/s10508-006-8993-y 16502152

[bibr35-0306624X221086569] Laws D. R. Marshall W. L. (1990). A conditioning theory of the etiology and maintenance of deviant sexual preferences and behavior. In Marshall W. L. Laws D. R. Barbaree H. E. (Eds.), Handbook of sexual assault: Issues, theories, and treatment of the offender (pp. 209–230). Plenum Press.

[bibr36-0306624X221086569] Leitenberg H. Henning K. (1995). Sexual fantasy. Psychological Bulletin, 117(3), 469–496. 10.1037/0033-2909.117.3.469 7777650

[bibr37-0306624X221086569] Longpré N. Guay J. P. Knight R. A. (2019). MTC Sadism Scale: Toward Dimensional Assessment of Severe Sexual Sadism. Assessment, 26(1), p.70-84. 10.1177/1073191117737377 (Online first, 2017). 29058955

[bibr38-0306624X221086569] Longpré N. Guay J. P. Knight R. A. Benbouriche M. (2018). Sadistic offender or sexual sadism? Taxometric evidence for a dimensional structure of sexual sadism. Archives of Sexual Behavior, 47(2), 403–416. 10.1007/s10508-017-1068-4 29204815

[bibr39-0306624X221086569] Longpré N. Sims-Knight J. E. Neumann C. Guay J. P. Knight R. A. (2020). Is paraphilic coercion a different construct from sadism or simply the lower end of an agonistic continuum? Journal of Criminal Justice, 71, 1–11.

[bibr40-0306624X221086569] Love T. Laier C. Brand M. Hatch L. Hajela R. (2015). Neuroscience of internet pornography addiction: A review and update. Behavioral Sciences, 5, 388–433. 10.3390/bs5030388 26393658 PMC4600144

[bibr41-0306624X221086569] Mellor L. (2017). Necrophilia-spectrum behavior and the thematic-derivative model of sexual progression. In Mellor L. Aggrawal A. Hickey E. W. (Eds.), Understanding necrophilia: A global multidisciplinary approach (pp. 203–220). Cognella.

[bibr42-0306624X221086569] Ogas O. Gaddam S. (2011). A billion wicked thoughts: What the Internet tells us about sexual relationships. Penguin.

[bibr43-0306624X221086569] Paolucci E. O. Genuis M. Violato C. (1997). A meta-analysis of the published research on the effects of pornography. Medicine, Mind and Adolescence, 72(1–2), 48–59.

[bibr44-0306624X221086569] Poulsen F. O. Busby D. M. Galovan A. M. (2013). Pornography use: Who uses it and how it is associated with couple outcomes. Journal of Sex Research, 50(1), 72–83. 10.1080/00224499.2011.648027 22449010

[bibr45-0306624X221086569] Ruscio J. Haslam N. Ruscio A. M. (2006). Introduction to the taxometric method: A practical guide. Lawrence Erlbaum.

[bibr46-0306624X221086569] Silver C. (2018). Pornhub 2017 year in review insights report reveals statistical proof we love porn. Retrieved April 3, 2019 from https://www.forbes.com/sites/curtissilver/2018/01/09/pornhub-2017-year-in-review-insights-report-reveals-statistical-proof-we-love-porn/?sh=2befa7ca24f5.

[bibr47-0306624X221086569] Warren J. Hazelwood R. (2002). Relational patterns associated with sexual sadism: A study of 20 wives and girlfriends. Journal of Family Violence, 17(1), 75−89.

[bibr48-0306624X221086569] Wells B. E. Twenge J. M. (2005). Changes in young people’s sexual behavior and attitudes, 1943–1999: A cross-temporal meta-analysis. Review of General Psychology, 9(3), 249–261.

[bibr49-0306624X221086569] Wilson G. D. Lang R. (1981). Sex differences in sexual fantasy patterns. Personality and Individual Differences, 2(4), 343–346.

